# Safer Systems: People Training or System Tuning?

**DOI:** 10.3390/ejihpe11030073

**Published:** 2021-08-31

**Authors:** Erik Hollnagel

**Affiliations:** Institute of Resilient Systems Plus, 40 Sogokro, Manangu, Anyangsi 14089, Korea; sensei@safetysynthesis.com

**Keywords:** system perspective, functional analysis, human factors, Safety-II

## Abstract

Safety is usually seen as a problem when it is absent rather than when it is present, where accidents, incidents, and the like represent a lack of safety rather than the presence of safety. To explain this lack of safety, one or more causes must be found. In the management of industrial safety, the human factor has traditionally been seen as a weak element; human error is often offered as the first, and sometimes the only cause of lack of safety and human factors have since the early days offered three principal solutions, namely training, design, and automation. Of these, training has considerable face value as an effective means to improve human performance. The drawback of safety training, however, is that it focuses on a single system component, the human, instead of on the system as a whole. Safety training further takes for granted that humans are a liability and focuses on overcoming the weakness of this specific component through simplistic models of what determines human performance. But humans may also be seen as an asset which changes the focus to strengthening how a complex socio-technical system functions. A socio-technical system comprises multiple functions that must be finely tuned in order to ensure expected and acceptable performance. Since systems cannot be made safer without developing effective ways of managing the conditions in which people work, system tuning offers an alternative solution to an old problem.

## 1. Introduction

To begin, consider a fictive dialogue between an industrialist (I) and an academic (A):

I: I have a problem with safety in my company. I want to improve it. Can you help me?

A: Perhaps, but I need to know what exactly you mean by safety.

I: I mean, of course, that there should be fewer or no accidents.

A: In that case, the solution is easy. Tell your people not to do anything.

I: That is no good, because I also want production to go on and something to be done.

A: Aha, but then your problem is not just safety and not the avoidance of accidents. It is rather how to make sure that work goes well—so you have production and as few accidents as possible.

I: Exactly, that is what I am looking for. How do I achieve that?

As this dialogue makes clear, it is important to know what the problem is before trying to find a solution. In practically everything that is said and written about safety, it is taken for granted that the outcomes can be described by binary terms, such as ‘safe’ and ‘unsafe’, or ‘success’ and ‘failure’—although there are far more terms for what happens when safety is absent than for when it is present. Safety—or rather, safe performance—has been associated with the absence of what is unwanted, with freedom from accidents and injury, at least since 1931 [1]. Since no sensible person can disagree with this objective, the presence of unwanted outcomes is tacitly accepted as the problem, and all efforts are directed at finding solutions to this problem. However, it may be worthwhile to first look more closely at what the problem is.

The binary nature of the outcomes of actions means that there in principle are two ways forward: either to reduce the number of unacceptable outcomes, or to increase the number of acceptable outcomes. Since something cannot fail and succeed at the same time, at least not in the macroscopic world that we live in, ensuring that everything goes well logically means that nothing goes wrong (The converse is unfortunately not the case). Increasing the number of acceptable outcomes will therefore bring about the same results as decreasing the number of unacceptable outcomes, but by means of a completely different approach. This can also be put as a question of whether the aim is to make performance less unsafe or whether it is to make it safer. The former corresponds to a Safety-I perspective, while the latter corresponds to a Safety-II perspective [2]. The former leads to a search for weak components or factors that can be eliminated, strengthened, or improved, while the latter creates a need to understand why and how work goes well, and to find ways to facilitate and amplify that.

## 2. The Legacy of Safety Training

Ever since Leucippus of Miletus declared that nothing happens in vain but that everything happens for a reason and of necessity, the principle of causality has been the universally accepted foundation for thinking about and dealing with the world we live in. This is not least the case when it comes to how safety professionals deal with the occurrence of unwanted events and the prevention of unwanted outcomes. Since unwanted outcomes are seen as a result of unsafe functioning or performance, the first priority is, not surprisingly, to find the causes of unsafe performance so that they can be eliminated.

In the 1970s, before the idea that human factors affect safety became prominent, accidents were mostly seen as being due to the uncontrolled transfer or release of energy [3]. At that time, systems and work situations were intelligible and the causes usually tangible. The steps taken to improve safety were therefore fairly straightforward. The first was to try to control and prevent the build-up of energy that could cause damage or harm. Follow that by creating work environments that are less likely to lead to such situations. And finally establish measures to counteract the injurious build-up of energy.

From the 1980s and onwards, accidents were with increasing frequency explained by invoking the human factor—by pointing to what people either failed to do or were judged to do incorrectly during their work. However, regardless of the changed frame of reference, the proposed solution remained the same, namely to eliminate or reduce the causes that supposedly could lead to unacceptable outcomes. In the case of human factors, the simple solution—to find the causes of human failures and select an effective remedy to prevent them—did not work well because socio-technical systems were different from technical systems and harder to understand. The causes were in many cases unfortunately intangible and the possible relations between causes and consequences were not only often hypothetical but also difficult to describe precisely.

The human factor had from the earliest days of human factors engineering been seen as something that could adversely affect system performance. One authoritative source defines the goals of human factors engineering as being to reduce error(s), increase productivity, enhance safety, and enhance comfort [4]. This was difficult enough when the focus was dyadic human–machine (or human–technology) interaction. Extending the scope from individual humans or operators to teams, and later on to organisations, only made the problem worse. Although it was still possible to explain events by invoking persuasive but hypothetical intervening variables or factors, such as safety culture, the intellectual satisfaction of naming a cause did not always lead to practical solutions. If, for instance, injuries (or harm) are due to or caused by the safety culture—or by some other hypothetical factor—then how can they be reduced or prevented?

### 2.1. Classical HF Solutions: Training, Design, and Automation

Human factors engineering came into being around 1945 because technological progress had enabled the construction of technological artefacts (i.e., machines) that were so fast and complex that humans were unable to control them effectively. This necessitated a solution, not only to lighten the burden on humans, but also to ensure that the performance of the new machines would not be encumbered by human shortcomings. From the beginning there were two obvious solutions on offer:

“It is now common to regard the human operator of a machine and the machine itself as two elements in one overall man-machine system. To improve the performance characteristics of these man-machine combinations, one has the choice of either trying to alter the man so that he fits the machine better, or modifying the mechanisms to fit the man.”

[5]

In other words, training or design. A third solution was to let machines do the tasks for which humans were ill-fitted, as expressed by the following famous statement:

“We begin with a brief analysis of the essential functions. We then consider the basic question: Which of these functions should be performed by human operators and which by machine elements?”

[6]

The first solution—training—was in line with the principles of Scientific Management that even now are widely applied, although with fewer of its original ideological overtones. The second solution, design, which began as human factors engineering but later morphed through ergonomics to become cognitive ergonomics, is also still in use and has through the years been embellished with various design philosophies, such as usability engineering or ecological interface design. The third solution opened the way for various types of automation and started a debate that is still raging [7,8]. All three solutions have thus been applied to solve safety problems over the years, although with varying degrees of success.

### 2.2. The Training Dilemma

Training was one of the four elements that characterised the practice of Scientific Management introduced in 1911 [9]. Although the focus was on productivity, only a slight change of words is needed to use the same arguments for safety. Consider, for instance, the first and second elements proposed by Taylor:

1. Develop a science for each element of a man’s work, which replaces the old rule-of-thumb method.
2. Scientifically select and then train, teach, and develop the workman, whereas in the past he chose his own work and trained himself as best he could.

Transposed to a safety context, the two elements might read like this:

1. Carry out a risk/safety analysis of work and develop the necessary procedures and guidance.
2. Train people to perform according to the procedures and guidance.

The first element is to identify the potential causes of problems (risks and hazards) as a basis for determining a safe way of carrying out the work. The second element corresponds to the contemporary principle of safety compliance, which can be defined as the state of being in accordance with established safety standards and regulations. But can it be taken granted, however, that training is an effective way to bring about desired performance?

One of the earliest studies of the effectiveness of training was published in 1959 in the second issue of the journal *Ergonomics*. It considered whether it was possible through operator training to adjust the human component to the requirements of the machine, and thereby eliminate performance differences among man–machine systems of different intrinsic merit [5]. Since safety had not yet become a major issue, the focus was on efficiency, or productivity. The study pointed to a serious dilemma over training as a solution, which was characterised by invoking the legendary case of Procrustes from Greek mythology. According to this legend, Procrustes was a rogue smith and robber from Attica who forced people to fit the size of an iron bed in one of two ways. If people were too short, they were stretched to become long enough; and if they were too long, they were made shorter by cutting off their feet or legs.

In the context of training Taylor and Garvey argued that people were “stretched” when they were trained to do more than was natural for them. Similarly, training could be used to curtail what people did by forcing them to comply with a strict and simplified procedure, hence making them “shorter”. This might work satisfactorily during normal working conditions. However, when people become stressed under unusual conditions, such as an accident, the effects of training break down and people revert to what is natural to them. If their performance has been “stretched”, they will end up not doing enough; and if their performance has been curtailed, they will end up doing too much. Taylor and Garvey concluded that the efficacy of training is unreliable in critical situations, even though it may work well enough in situations that are not stressed.

## 3. The Meaning of Safety

Another important consideration is, of course, the meaning of the word safety in safety training. It has been pointed out that “safety is defined and measured more by its absence than by its presence” [10]. Therefore, safety can either be defined as a condition of being without harm, or other unacceptable outcomes (Safety-I), or as a condition of being with intended and acceptable outcomes (Safety-II) [2]. The choice of definition will inevitably have consequences for the role and purpose of training. In the first case, the aim is to avoid the occurrence of unwanted situations occur; in the second, it is to ensure that desired situations occur.

In practically all writings about safety training, the implicitly accepted definition of safety is the first rather than the second. This is obvious from how safety is measured in the workplace, typically by the rate of near misses, injuries, illnesses, and fatalities. However, by focusing on negative outcomes, safety is measured by its absence rather than its presence. The purpose of safety performance should obviously be to avoid these outcomes rather than to produce them. Safety training should therefore include appropriate measures and practices that can contribute to preserving the life and health of individuals as well as the integrity of the workplace. This is illustrated by a model that is typical of current research.

**Figure 1:**
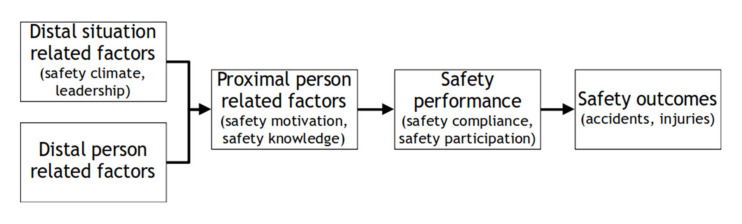
An integrative model of workplace safety (adapted from [11]).

The model purports to show the relation between safety performance and safety outcomes, described as accidents and injuries. Although no definition is provided of what training is or entails, the model in Figure 1 shows four commonly used factors or antecedents, namely safety motivation, safety knowledge, safety compliance, and safety participation. Safety performance is seen as a combination of the latter two.

One problem with this definition of safety is that, from a learning perspective, it is notoriously difficult to train for something that is defined by its absence, to train to avoid doing something. It is much easier and more effective to train for acceptable performance, to train to do something well. This therefore leads to a question over what is meant more precisely by safety performance, and subsequently the question of what determines or shapes such performance.

### 3.1. Safety Performance

Changing or improving safety performance is easier said than done. Psychology has throughout its history entertained many different theories about what determines performance, with behaviourism, dynamic psychology, and field theory being prominent examples. In relation to safety, the notion of behaviour-based safety (BBS), loosely defined as the application of behaviour change techniques [12] to real world safety problems, is very popular. (Since the term *behaviour* is notoriously difficult to define, the term *performance*, meaning “what a person does when faced with a task” [13] shall be used instead.) This implies that there are two clearly distinguishable categories of human performance, one being unsafe, or risky, and the other not. It also implies that specific training techniques can be used to suppress the former and that others can be used to enhance the latter, and that the causes of and consequences for either category have the same valence. (In other words, that negative or unacceptable consequences such as accidents are due to negative or unsafe performance, while positive or acceptable consequences, meaning work that goes well, are due to positive performance.)

It is, however, questionable whether this assumption is correct. In the first book on resilience engineering it was argued that “failure is the flip side of success, and therefore a normal phenomenon” [14]. This was hardly new, since Ernst Mach more than a century earlier had made exactly the same point when he noticed that “knowledge and error flow from the same mental sources, only success can tell one from the other” [15].

The acceptance that people usually try to do what makes sense to them in a given situation—and how could they do anything else?—still leaves unanswered the question of what actually determines performance in general and safety performance in particular. Are the proximal factors, to use Brunswik’s [16] term, the most important so that safety performance can be shaped more or less directly? Or are the distal factors more important, so that safety performance is determined by culture and background rather than the actual work environment and situation? Should safety training look at these manifestations and try to change them directly, as in BBS, with no concern for what may lie behind? Or should safety training rather look at the more abstract factors hypothesised as the basis for performance, either the monolithic concept of safety culture [17] or a more detailed description such as, for example, the model of organisational culture proposed by Edgar Schein [18]? If performance, or to use a contemporary term, Work-as-Done, is seen as being influenced by proximal and/or distal factors rather than occurring in isolation, as a hostile *deus ex machina*, then these influences must surely be the same for all situations rather than differ between safe and unsafe performance. However, if that is the case, then it makes little sense for safety training to focus on avoiding something, on eliminating certain types of performance.

(As a brief terminological note, safety performance or safety behaviour seem to confuse actions with outcomes. Instead of “safety performance” it would be better to describe the action as, say, “performance in safety-critical conditions”, and then characterise what these conditions are. For instance, that accident prevention is the top priority, that uncertainty is increased, that time is limited, that the work environment is degraded, etc. Alternatively, “safety performance” could be substituted by a reference to the outcome, such as “performance with acceptable outcomes”.).

### 3.2. What Determines How People Perform?

What do people do when faced with a task? By definition, acting or doing something always takes place in a context, in a situation or a set of conditions about which it is impossible to know everything ahead of time (the Work-as-Imagined versus Work-as-Done dilemma, cf. [19]). Neither do people exposed to these situations function as simple stimulus-response organisms or programmable machines, to use different analogies. A plethora of studies in psychology, social psychology, and organisational behaviour has shown that people are not rational machines but that as a rule, they adjust what they do to fit the situation. Famous examples are studies and analyses of administrative behaviour [20], of judgment under uncertainty [21], and of naturalistic decision-making [22]. Human performance is never a simple result of a few factors, as Figure 1 implies. Accounting for the intricacies of human performance is therefore not an easy undertaking. This was illustrated by a performance model described by McCloy et al. [23], which defined job performance as a function of knowledge of facts, rules, principles, and procedures, more precisely as the capability attained when the knowledge of what to do is successfully combined with knowledge about, and the ability, to do it. It was expressed both in a formula, as PC = (DK, PKS, M), and in words, as follows:

“Simply stated, the hypothesized performance function indicates that to perform a task, a person must (a) possess the prerequisite knowledge, (b) master the prerequisite skills, and then (c) actually choose to work on the job tasks for some period of time at some level of effort.”

(McCloy et al., p. 494)

But how can training effectively improve this? Since the working conditions never will be exactly as imagined [19], neither the prerequisite knowledge nor the prerequisite skills can be described precisely in advance. Moreover, standardising working conditions is not an effective solution [24], even though it may be needed to complement training. It is also left open how training can contribute to make a person “*work on the job tasks for some period of time at some level of effort*”. Since the target of training is non-trivial, training will require that a combination of methods and interventions are applied at the same time. A survey of 133 studies identified nine different techniques for safety training [25]. Similarly, others have found that training interventions typically involve multiple methods, such as lectures, printed materials, hands-on practice, and feedback [26]. To complicate the picture even further, other researchers [27] have described how seven other factors, such as group size, trainer qualifications, and management role, can have an effect on how effective safety training is. In the same vein, it has been suggested that a combination of micro and macro organisational factors can affect safety compliance and, therefore, safety performance [28]. If these concerns are added to the brittleness of performance pointed out by Taylor, et al. [5], it seems doubtful that safety training actually can be an effective solution, just as it is hard to see how it works and to know whether it will work.

## 4. How Can Systems Be Made Safer?

Safety training at first looked like a simple solution to a simple problem. The simple problem was that human performance was assumed to be potentially unsafe and the main cause of unwanted consequences or outcomes. The simple solution was therefore to improve safety performance—or rather, to improve human performance in safety-critical situations. This way of reasoning followed the tradition of breaking bigger problems into smaller problems and then solving these by one means or another. Human factors engineering is an almost paradigmatic illustration of this. The three traditional solutions—training, design, and automation—are based on decomposing systems into their main constituents: humans, machines, and the interaction between them. Training is used to “improve” the human part, design is used to “improve” the interaction, and automation is used to “improve” the machines so that they can replace the humans. But the problem has turned out to be complex rather than simple. And whereas simple problems may have simple solutions, complex problems do not. Disguising complex problems as simple problems by offering apparently “simple” solutions does not really make the problems any simpler. It only makes it more likely that the solutions will not work.

The alternative to avoiding the complexity of actual work situations by decomposing systems into parts and problems into sub-problems is to look at systems as aggregated wholes and to characterise problems on that level. In 1983, Cognitive Systems Engineering proposed the principles for coping with complexity [29], borrowing a term used by Rasmussen and Lind [30]. From this perspective, attempts to improve the performance of a system by focusing on the parts and improving these one-by-one ignores the dependency among the parts. This might have been justifiable sixty years ago, when it was reasonable to think in terms of human–machine systems, but it is no longer the case. The systems of today are socio-technical systems and complex ones at that, where the interrelation or dependency among system functions is often more important than the reliability of the parts. Improving safety performance must therefore be based on an understanding of what happens in the system, of the nature of its interactions and couplings [31], and of how its overall performance can be managed and improved.

### 4.1. Training or Tuning?

From a joint-systems perspective it is still useful—and practically necessary—to describe systems in terms of their details or particulars, but the focus should be on functions and how they can be made to work together. To achieve desired performance, and to develop a training programme for that, it is necessary to consider which skills and competences are required for the chosen purpose and to keep in mind that people’s performance always serves multiple purposes and never the avoidance of accidents alone. Whatever is trained must therefore be synergistic with everything else, lest performance become fragmented and degraded by different (and competing) foci. This is consistent with the advice to “[r]eject countermeasures focused on individual behaviour” [32]. The recommendation is to look instead at the complex of interventions that constitute responses to system-wide issues. The solution, in other words, is to tune functions to match each other, rather than to optimise—through training or by other means—the isolated performance of a few of them.

Tuning is an engineering term that describes how the functioning of a system can be optimised for a particular environment by adjusting critical parameters and functions. One may thus “tune for time” to perform faster, “tune for space” to reduce demands on storage capacity, or “tune for configuration” to obtain the most efficient use of available resources. Lean manufacturing can be seen as a form of tuning whose purpose is to reduce time within the production system, as well as response times to customers. In the same vein, it is possible to tune for safety, which today means adjusting the system’s safety performance to ensure that as much as possible goes well.

Whereas training is based on a trivial structural view, according to which a system is understood to be composed of parts or elements, tuning is based on a non-trivial functional view, according to which a system is seen as an organised set of functions that together will achieve the purpose. Although the ability to do so in practice may be ascribed to any number of “management skills”—such as planning, communication, decision-making, the ability to motivate, etc.—it is clearly not enough. It is also necessary to have a more articulate understanding of how complex socio-technical systems function, within which management is just one function among others. This requires a systematic approach to identifying essential functions and how they are mutually dependent or coupled [33]. Just as the complexity and non-linearity of these couplings can be seen as the origin of “normal accidents” [31], their effective management can be seen as a prerequisite for acceptable system performance, not just with regard to safety but across multiple criteria. While it is valuable to understand how stable performance can be disrupted by internal and external events, there is also a need better to understand, analyse, and describe how stable functioning can be established or sustained. The solution to the safety problem is therefore to develop effective ways to manage the conditions in which people work rather than to change the human condition.

### 4.2. Final Dialogue

A: Did this make sense to you?

I: I think so. I now understand that the problem is not simply to train people to work more safely. The problem is rather to find ways to improve—or as you say, to tune—how my company works and to understand how this happens. But how can I do that?

A: One way to start is to identify the functions that are essential for the performance you want.

I: Oh, that is easily done. We have that already in the description of our organisational roles and responsibilities.

A: Well, actually, you should look at what actually happens, at how work is done, and not at how work is imagined.

I: I see. So I should find out how my company operates when nothing goes wrong, when safety is present?

A: Exactly. So now let us look at how this can be done ……
